# Active auditory experience in infancy promotes brain plasticity in Theta and Gamma oscillations

**DOI:** 10.1016/j.dcn.2017.04.004

**Published:** 2017-04-15

**Authors:** Gabriella Musacchia, Silvia Ortiz-Mantilla, Naseem Choudhury, Teresa Realpe-Bonilla, Cynthia Roesler, April A. Benasich

**Affiliations:** a Center for Molecular and Behavioral Neuroscience, Rutgers University-Newark, 197 University Ave., Newark, NJ 07102, United States; b School of Social Science and Human Services, Ramapo College of New Jersey, 505 Ramapo Valley Rd., Mahwah, NJ 07430, United States

**Keywords:** Auditory, EEG, Development, Brain oscillations, Infant, Plasticity

## Abstract

Language acquisition in infants is driven by on-going neural plasticity that is acutely sensitive to environmental acoustic cues. Recent studies showed that attention-based experience with non-linguistic, temporally-modulated auditory stimuli sharpens cortical responses. A previous ERP study from this laboratory showed that interactive auditory experience via behavior-based feedback (AEx), over a 6-week period from 4- to 7-months-of-age, confers a processing advantage, compared to passive auditory exposure (PEx) or maturation alone (Naïve Control, NC). Here, we provide a follow-up investigation of the underlying neural oscillatory patterns in these three groups. In AEx infants, Standard stimuli with invariant frequency (STD) elicited greater Theta-band (4–6 Hz) activity in Right Auditory Cortex (RAC), as compared to NC infants, and Deviant stimuli with rapid frequency change (DEV) elicited larger responses in Left Auditory Cortex (LAC). PEx and NC counterparts showed less-mature bilateral patterns. AEx infants also displayed stronger Gamma (33–37 Hz) activity in the LAC during DEV discrimination, compared to NCs, while NC and PEx groups demonstrated bilateral activity in this band, if at all. This suggests that interactive acoustic experience with non-linguistic stimuli can promote a distinct, robust and precise cortical pattern during rapid auditory processing, perhaps reflecting mechanisms that support fine-tuning of early acoustic mapping.

## Highlights

• Active acoustic experience (AEx) in infancy impacts cortical oscillations.
• AEx infants show left Theta- and Gamma-band activity to complex tone pairs.
• Passive and naïve infants yield less distinct, more bilateral responses.

## 1 Introduction

While several peripheral auditory functions appear to be adult-like at birth, developmental changes in the upper brainstem and auditory cortex continue over several years (Kinney et al., 1988, Paus et al., 2001, Deoni et al., 2011). Plasticity related to temporal-processing speed, including synaptic proliferation, pruning and the development of axonal myelin sheaths (Su et al., 2008) comprise such a trajectory. Maturation of efficient processing of spectro-temporal acoustic cues is critical for perception of complex sounds, the establishment of phonemic representations, and for normative speech discrimination (Aslin, 1989, Khul, 2004, Tallal and Gaab, 2006). If difficulties in processing such rapidly-presented sounds arise, they are highly predictive of concurrent and later language abilities (Benasich, 2002, Benasich and Tallal, 2002, Tallal et al., 2004, Choudhury and Benasich, 2011). Therefore, understanding the timeline and mechanisms underlying temporal-processing development in the maturing auditory system is of considerable importance, both from basic science and clinical application standpoints.

Recent event-related potential (ERP) research from our lab suggests that early, targeted acoustic experience can enhance and accelerate the maturation of temporal processing in typically-developing infants (Benasich et al., 2014). In an auditory discrimination experiment, infants who received acoustic exposure to rapidly-presented tone pairs between 4- and 7-months-of-age had more mature ERP waveforms compared to age-matched naïve controls. Further, infants who experienced interactive, real-time feedback showed enhanced temporal sensitivity, more efficient/faster processing of key acoustic cues and greater generalization to untrained sounds, compared to infants who passively listened to the same sounds. These findings suggest that interactive participation in an acoustic experience may be a powerful catalyst for promoting temporal sensitivity beyond passive listening or maturation alone.

Key mechanistic questions arise from these results, including where in the brain these changes take place and what neurophysiological mechanisms support these modifications. Classical analysis of scalp-recorded ERP data blur such response features because the peak latency and amplitude of channel-based ERP data sum across space, frequency, phase and magnitude (Baser et al., 2011). Determination of the spatial location of sources that give rise to ERP responses can be accomplished by estimating the underlying current dipoles in space and time [e.g. (Gloor, 1985)]. Such analysis can approximate activity with a resolution of ∼100 cortical cells (Nunez, 1981). Time-frequency analysis of single trials of evoked activity yields a deeper understanding of brain responses via measures of power magnitude and phase-coherence across events (Makeig, 1993). Because brain responses often involve activity in one or more discrete frequency bands, full-spectrum analysis [e.g. Delta (1–4 Hz), Theta (4–8 Hz) to Gamma (>25 Hz] also gives additional specificity compared to broadband ERPs.

The onset of an auditory stimulus resets the phase of neuronal oscillations in auditory cortex, which often co-occurs with the discharge of an action potential. Event-related oscillations in the mature system have been characterized by two phenomena: nested phase-locking and asymmetry of temporal processing. In nested phase-locking, evoked oscillations in the lower frequency bands of Delta and Theta synchronize to the slower temporal dynamics sound such as the speech envelope (Abrams et al., 2009, Giraud and Poeppel, 2012, Luo and Poeppel, 2012) while fast oscillations in the Gamma frequency range are associated with the encoding of rapid feature analysis, temporal binding of stimulus events and attention control (Steriade et al., 1990, Pantev, 1995, Fries et al., 2007). Event-related oscillations have been shown to lateralize asymmetrically as a function of time scale, with low-frequency activity encoding slow auditory fluctuations, primarily processed in right auditory cortex (RAC) and high-frequency activity encoding rapid auditory changes, predominant in left auditory cortex (LAC) (Poeppel, 2003). The phenomenon of temporal asymmetry does not appear to be restricted to event-related oscillations or adults, as a recent study showed a leftward cortical bias in 3–5 year olds for fast, speech-related frequency phase-locking in spontaneous oscillatory activity (Thompson et al., 2016).

Our previous studies show that tone and phoneme discrimination in infancy evoke activity in both Theta and Gamma bands (Musacchia et al., 2013, Ortiz-Mantilla et al., 2013), but the degree to which these activity patterns are age-, hemisphere- or exposure-dependent remains unclear. As targeted acoustic experience in early infancy has been shown to accelerate maturation of temporal auditory processing and acoustic mapping (Benasich et al., 2014), we were interested in examining differences in Theta and Gamma oscillatory patterns as a function of such experience. To do this, we performed spatial and time-frequency analysis of auditory maturation and exposure-related brain plasticity in infants using the electrophysiological data reported in Benasich et al. (2014) and investigated the specific characteristics of neuronal oscillations as a function of interactive or passive exposure to non-linguistic, temporally-modulated stimuli. We hypothesized that infants who received either passive exposure (PEx) or interactive auditory experience (AEx) with these temporally-modulated stimuli between 4- and 7-months-of-age would differ in patterns of oscillatory dynamics from 7-month-old naïve infants (NC) without such experience. Based on previous data, differences in the magnitude and specificity of temporal spectral evolution (TSE) in the frequency oscillations observed during rapid tone discrimination were predicted. The TSE value captures induced (random-phase/non-phase-locked) and evoked (phase-locked) event-related changes in the amplitude of oscillatory activity in response to stimulus presentation (Hoechstetter et al., 2004). In particular, we posited that the enhanced auditory processing efficiency observed in the AEx group might be supported by left-lateralized (i.e. more mature) patterns of oscillatory activity in higher frequency bands as compared to the PEx or NC groups.

## 2 Materials and methods

Methods for recording and analysis in the current study follow those in previous publications from our laboratory (Hämäläinen et al., 2011; Ortiz-Mantilla et al., 2012; Musacchia et al., 2013; Ortiz-Mantilla et al., 2013; Benasich et al., 2014; Musacchia et al., 2015). Abbreviated versions of these methods are provided below.

### 2.1 Participants

**Fig. 1:**
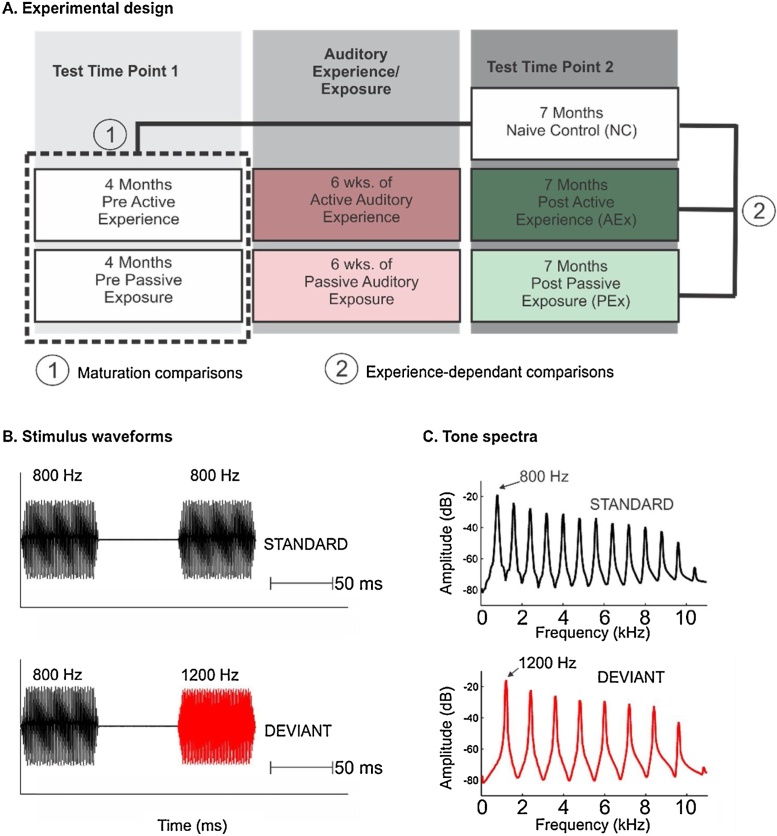
Schematic of overall study design and stimuli. (A) Infants were tested at two time points (4- and 7-months-of-age) with either Passive Auditory Exposure (PEx) or Active Auditory Experience (AEx) between the two sessions. Naïve 7-month-olds were recruited without prior lab testing (NC). To examine maturational effects, statistical tests were calculated with naïve 4- and 7-month-old measures. The three 7-month-old groups were compared to delineate significant experience-related effects. (B) Stimulus waveforms. Complex tone pairs were presented in a passive oddball paradigm using a blocked design. (C) Tones had a fundamental frequency of 800 or 1200 Hz with 15 harmonics (6-dB roll-off per octave).

This study was conducted in accordance with the Declaration of Helsinki. Informed consent, approved by the Institutional Review Board of our university, was obtained from all parents before study participation. Parents were compensated for their time, and infants received a toy after the visit. A total of forty-nine infants participated in this study. Infants were from monolingual English families with no reported family history of specific language impairment or of dyslexia, learning disability, attention deficit disorder, pervasive developmental disorder, or autism in either the nuclear or extended family (grandparents, aunts and uncles). Before the first testing session, each infant was randomly assigned to one of three groups (Benasich et al., 2014). Fig. 1A illustrates the study design, which comprises both maturational and experience-dependent comparisons. Two of the three groups were tested pre-exposure as naïve infants at 4-months of age (*m* *=* 18.2 weeks, *SD* = 0.77) and then followed longitudinally to 7-months-of-age (*m* = 31.0 weeks, *SD* = 0.65). The Active Experience group (AEx, n = 18, 10 males) participated in operantly-conditioned feedback-modulated auditory training (e.g. Benasich et al., 2002; Choudhury et al., 2007) between testing sessions and the Passive Experience group (PEx, n = 17, 9 males), were exposed passively and without feedback, to the same auditory training signals over the same time interval (see next section for training paradigm and stimulus description). The third group was recruited at 7-months-of-age (*m* = 30.6 weeks, *SD* = 1.54) and served as “naïve”, cross-sectional, maturational controls (NC, n = 14, 7 males).

### 2.2 Active auditory exposure

Infants in the AEx group visited the lab once a week for six consecutive weeks between 4 and 7 months of age. Each session lasted approximately 20 min with infant active engagement lasting about 8 min. The AEx group participated in a go/no-go (G/N-G) operantly-conditioned looking task designed to train an association between an auditory stimulus and the onset of a video reward (Nawyn et al., 2007). The procedure followed three phases: Familiarization, Training, and Baseline. During all phases, a standard stimulus was repeatedly presented, interspersed with an experimenter-initiated target stimulus paired with a video reward presentation. During Familiarization, the target stimulus was non-contingently paired with the reward video for up to 10 presentations. During the Training phase, the reward video was presented if the infants directed their gaze to a specified reward region on a computer screen in a “go” trial. The reward video was initiated automatically via eye-tracking software when the infant looked toward the reward area at any point over the “go” trial window. The training phase ended when the child responded correctly to 3 of 5 successive “go” trials or when a total of 10 target trials were presented. The child then proceeded to Baseline phase, which consisted of 10 “go” trials and 10 “no-go” trials. The sound stimuli were presented at varying inter-stimulus-intervals (ISIs) using an up-down staircase procedure (Trehub et al., 1986). Thus, the ISI for each block of stimuli was increased or decreased according to infant performance (i.e. made progressively more difficult for correct responses, easier for an incorrect or no-response trial). Successful completion of the Baseline phase required the infant to show four of five correct responses on two “go” and two “no-go” trials within a block of five successive trials. All AEx infants (100%) were successfully able to learn the task and demonstrate contingency learning of the go/no-go procedure. The task continued for approximately 7–9 min each session, contingent on when the child fatigued or fussed. For a more detailed explanation of the AEx procedure, please refer to Benasich et al. (2014).

### 2.3 Passive auditory exposure

Infants in the PEx group also visited the lab once a week for six consecutive weeks between 4 and 7 months of age. Each session lasted 20 min as the infant sat comfortably in an infant seat placed on a chair equidistant between left and right speakers in a sound-attenuated and electrically shielded sound booth (Industrial Acoustics Company). The PEx group was exposed to the same stimuli as the AEx group. However, there was no response-contingent reward for PEx infant looking behavior (i.e., no looking response was required as this was a passive task). Sounds were presented free-field while the infant was silently entertained with puppets/silent toys to maintain alertness. Two blocks of stimuli were presented in random order at each session, 10 min at 40 ms ISI and 10 min at 70 ms ISI. This condition was designed to increase spectrotemporal processing efficiency through passive background auditory exposure.

### 2.4 Stimuli for active and passive experience protocols

Infants in both groups were presented with three different types of paired acoustic stimuli, as follows: weeks 1 and 2, complex tones (STD, 800–800 Hz; DEV, 800–1200 Hz); weeks 3 and 4, bandpass noise (STD: 400–1900 Hz and 400–1900 Hz; DEV: 400–1900 Hz and 800–1900 Hz); and weeks 5 and 6: simple sweeps (STD: 1600–1200 Hz and 1600–1200 Hz; DEV: 1600–1200 Hz and 1200–1600 Hz).

### 2.5 EEG stimuli and recording paradigm

70 ms complex tone pairs were presented in a passive oddball paradigm using a blocked design with an inter-stimulus interval of 70 ms and an inter-trial interval of 915 ms (Fig. 1B). Each tone was comprised of its fundamental plus 15 harmonics (Fig. 1C). Stimuli were generated with Goldwave computing software (St. John’s, NL Canada). An invariant-frequency tone pair was standard (708 tokens, STD, 85%, F0 = 800 Hz) and a tone pair with frequency change was deviant (125 tokens, DEV, 15%, F0_1_ = 800 Hz, F0_2_ = 1200 Hz). Presentation of DEV stimuli was pseudo-randomized with at least three and no more than 12 STD pairs presented before each DEV pair. All stimuli were presented using E-Prime software (Psychology Software Tools, Inc.) amplified (Furman Sound, Petaluma, CA) to a calibrated level of 56.1 dB sound pressure level (SPL). Sounds were presented in free-field to the infants via left and right speakers which were attached to opposite walls of a sound-attenuated and electrically-shielded sound booth (Industrial Acoustics Company, Bronx, NY).

Infants were seated on their caregiver’s lap in a comfortable chair, positioned with its center equidistant (30 inches) from the face of each speaker. An experimenter, present in the room, engaged the infant’s attention with a silent puppet show or other silent toys to help minimize their movement. Age-appropriate movies or cartoons were also played silently on a video monitor in front of the children. High-density EEG data were recorded from a 128-channel geodesic sensor, vertex-referenced net using an EGI (Electric Geodesic, Inc., Eugene, Oregon) recording system. The EEG was sampled at 250 Hz and bandpass filtered online at 0.1–100 Hz. For visualization of the net application and EEG recording with infants, see (Musacchia et al., 2015). Stimulus triggers were marked and exported offline to a MATLAB (MathWorks, Natick, MA) compatible format, using Net Station software (Electric Geodesic, Inc., Eugene, Oregon).

### 2.6 ERP data processing

EEG data were processed according to previously published parameters using BESA 5.3 Research (MEGIS Software GmbH, Gräfelfing, Germany), EEGLAB (Delorme and Makeig, 2004), ERPLAB (UC-Davis Center for Mind & Brain) and MATLAB environments (Musacchia et al., 2013, Musacchia et al., 2015). Noisy channels in the EEG were interpolated in EEGLAB using the spherical spline interpolation. Myogenic components in the continuous data were visually selected and rejected based on all of the following criteria: (i) a moderately small and clustered distribution on the topographic maps focused in electrodes following scalp musculature, (ii) short periods of high-frequency activation across channels and (iii) large amplitude fluctuations signaling disturbances of the electrode net. Continuous data were then filtered with a 1–15 Hz bandpass and epoched with a −1500 to 1500 ms window around stimulus onset. This epoch window was chosen to allow for analysis of low-frequency time periods (e.g. 2 Hz). An artifact rejection criterion of ±500 μV was applied to the epoched data and clean epochs were averaged to create STD and DEV individual ERP averages. STD and DEV grand averages were then created for each group by averaging the individual ERP waveforms per condition.

### 2.7 Source localization

The surface ERP was transformed into brain source activity, using BESA’s MRI co-registration and source montage approach (Scherg and von Cramon, 1986, Berg and Scherg, 1994). First, each individual’s STD average was co-registered with our 6-month-old average MRI template and age–appropriate head parameters in a 4-shell ellipsoidal head model. The distributed source model was then calculated, using the CLARA method (Classic LORETA Recursively Applied; MEGIS Software GmbH, Gräfelfing, Germany), to visualize the extent of generator activity (Hoechstetter et al., 2010). Based on the CLARA solution, previous literature in adults (Scherg and von Cramon, 1985, Scherg and von Cramon, 1986) and our laboratory’s previous infant tone data at 6 months-of-age (Hämäläinen et al., 2011), 2 free dipoles were fit over a window of +/−20 ms around each individual’s P1 peak. Dipoles localized to left and right temporal regions for 100% of the subjects. Following the dipole fit, amplitude and latency of the P1 and N1/N2* peaks of the left and right source waveforms were recorded in the STD (P1, N1) and DEV (P1, N2*) conditions (Table 1, Table 2). Peaks reported here were identified as a positive or negative deflection from baseline and were labeled according to their order of appearance (e.g., P1, N1, P2, and N2). The change discrimination peak (N2*) is defined as the latency of the negative peak for the deviant wave that indicates the beginning of the discrimination response (for further discussion, see Choudhury and Benasich, 2011, Benasich et al., 2014). As a final step, the 2-dipole model for each individual was applied as a fixed spatial filter onto that individual’s raw EEG data (Scherg and Ebersole, 1994), thus creating continuous Left Auditory Cortex (LAC) and Right Auditory Cortex (RAC) data for further time-frequency analysis.

**Table 1:** Peak Latency and Amplitude in the Left and Right Auditory Sources of Naïve 4- and 7-month-old Infants.

Condition-Source	P1 latency (ms)				N1/N2* Latency (ms)				N1/N2* Amplitude (μV)			
	4 months		7 months		4 months		7 Months		4 Months		7 Months	
	*M*	*SD*	*M*	*SD*	*M*	*SD*	*M*	*SD*	*M*	*SD*	*M*	*SD*
Standard-Right	204.0	*27.1*	175.7*	*24.0*	324.6*	*39.1*	331.7	*25.7*	−10.7*	*9.9*	−15.4*	*6.5*
Deviant-Right	209.2	*30.4*	174.6*	*26.5*	292.8*	*27.6*	294.5	*28.3*	−14.0*	*11.3*	−11.7*	*13.4*
Standard-Left	211.1	*27.2*	171.1*	*22.6*	327.5*	*35.8*	331.7	*29.5*	−6.4	*7.4*	−15.9	*10.4*
Deviant-Left	210.5	*31.8*	175.4*	*24.4*	301.8*	*27.3*	296.3	*34.3*	−9.7	*9.7*	−9.7	*8.3*

* p < 0.05; M: mean; SD: standard deviation; ms: milliseconds; μV: microvolts.

**Table 2:** Peak Latency and Amplitude in Left and Right Auditory Sources of 7-month-old Infants with Varied Auditory Experience.

Cond.-Source	P1 Amplitude (μV)			N1/N2* Latency (ms)			N1/N2* Amplitude (μV)		
	NC	PEx	AEx	NC	PEx	AEx	NC	PEx	AEx
	*M(SD)*	*M(SD)*	*M(SD)*	*M(SD)*	*M(SD)*	*M(SD)*	*M(SD)*	*M(SD)*	*M(SD)*
STD-R	18.1(3.5)	17.8(7.6)	18.8(7.9)	331.7(25.7)	328.0(46.5)	328.0(46.5)	−15.4(6.5)	−15.7(9.9)	−12.8(8.8)
DEV-R	18.9(6.3)	14.3(8.9)	19.1(12.1)	294.5(28.3)*	269.6(45.1)*	274.0(31.4)	−11.7(13.4)*	−13.0(6.5)^*^	−5.9(9.7)^*^
STD-L	17.5(6.4)	14.1(6.2)	15.1(6.1)	331.7(29.5)	332.0(42.0)	331.8(42.6)	−15.9(10.4)	−9.5(9.0)	−10.1(6.9)
DEV-L	15.1(7.2)	12.5(5.5)	17.3(8.3)	296.3(34.4)*	276.3(46.2)*	281.3(42.6)	−9.7(8.3)*	−9.1(9.5)^*^	−4.7(10.4)^*^

* p < 0.05; Cond: condition; STD: Standard; DEV: Deviant; R: right; L: left; ms: milliseconds; NC: Naïve Controls; PEx: Passive Exposure; AEx: Active Experience; M: mean; SD: Standard Deviation; μV: microvolts.

### 2.8 Time-frequency analysis

Instantaneous amplitude and phase to a three-step complex demodulation algorithm was applied to the continuous data in source space. Frequencies between 2 and 50 Hz were analyzed in 50 ms time bins, relative to a baseline pre-stimulus epoch of −100 to 0 ms (Papp and Ktonas, 1977, Hoechstetter et al., 2004). The demodulation provides measures of all brain activity which is both stimulus phase-locked and non-phase-locked. Two measures were used to assess time-frequency fluctuations. (1) Temporal spectral evolution (TSE) was used to examine event-related changes in amplitude (power) of the different frequency bands relative to the baseline (Tallon-Baudry et al., 1996, Hari and Salmelin, 1997). The TSE value represents the percentage of amplitude change of induced (random-phase/non phase-locked) and evoked (phase-locked) oscillatory activity related to stimulus presentation. (2) Inter-trial phase locking (ITPL) was used to measure how consistently the phase at different frequency bands locks to stimulation presented across trials. Its values range from 0 to 1; where 0 indicates random phase across trials and a value of 1 corresponds to perfect inter-trial phase alignment (Tallon-Baudry et al., 1996, Tallon-Baudry and Bertrand, 1999).

**Fig. 2:**
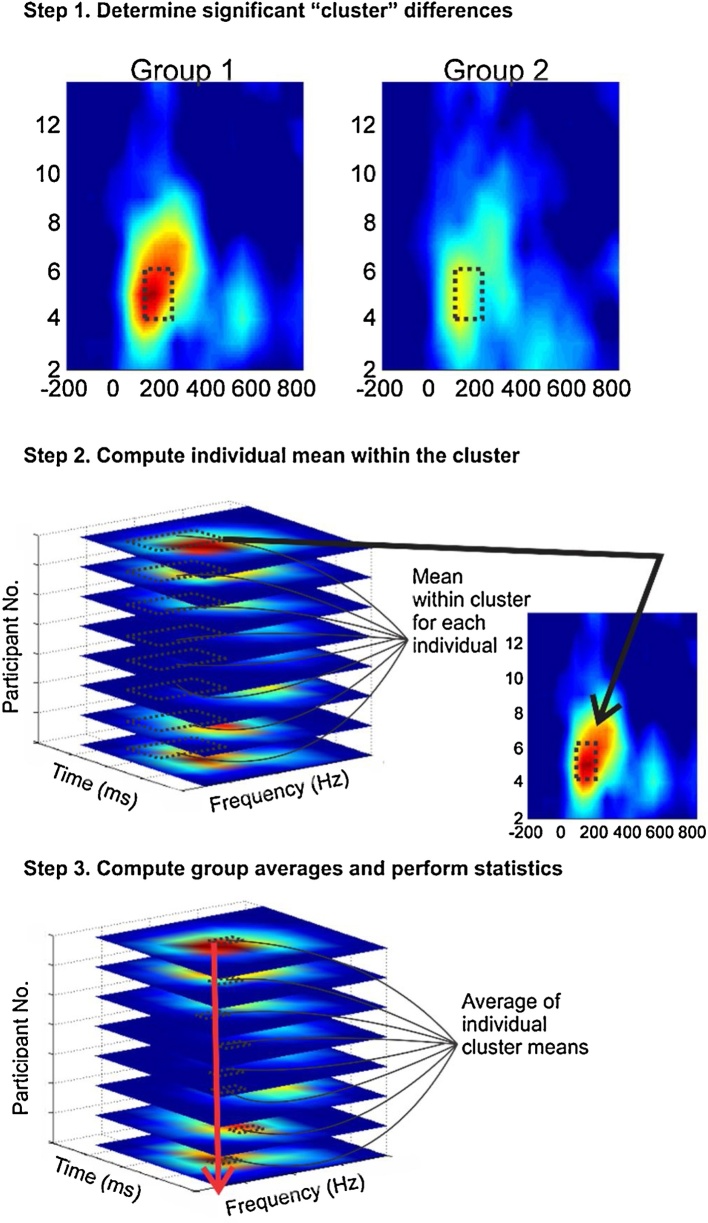
Determination of significant time-frequency “clusters”. Step 1. Significant and stable difference in “clusters” of time and frequency are automatically identified between groups. This figure shows an illustration of individual data matrices to be submitted to statistical analysis. Step 2. The mean value was then calculated within the cluster boundary for each individual. Step 3. A “subcluster” mean was then computed to give the individual data points for ITPL and TSE statistical analysis.

Mathematically, the time-frequency computation yields two 3-D matrices, for ITPL and TSE values, per participant (x: time, y: frequency, z: ITPL or TSE values). In order to obtain meaningful values for our planned statistical comparisons, we performed a data-driven, three-step analysis using these matrices. First, we detected regions of significant TSE and ITPL differences for the DEV response using BESA Statistics 1.0 (BESA, GmbH) software. This program calculates a preliminary Student's *t-test* between groups per data point. Second, the *t*-test values were submitted to parameter-free permutation testing, in combination with what is referred to as “data clustering”, in order to reduce type I error via multiple comparisons (a complete description of the method used can be found in the BESA Statistics Manual, 2011). Results of this analysis are described below and an example cluster is illustrated in Fig. 2 Step 1. Third, individual mean ITPL and TSE values were created by averaging each subject’s data matrix within the significant cluster (Fig. 2 Step 2). The results of this analysis yielded mean ITPL and TSE values for each individual (Fig. 2 Step 3).

Statistical analysis of the time-frequency matrices yielded three significant time-frequency clusters (p < 0.05):(1) Theta ITPL from 4 to 6 Hz over 150–250 ms, (2) Theta TSE from 3 to 7 Hz over 50–450 ms, and (3) Gamma TSE from 33 to 37 Hz over 200–500 ms. Individual means used in subsequent analyses were derived from within these cluster boundaries (Fig. 2).

### 2.9 Statistical analyses of brain response measures

A series of ANOVA models were conducted to assess the typical maturational changes from 4 to 7-months of age as well as the effects of auditory exposure over and above that of maturation on LAC and RAC peak latency and amplitude (P1 and N1/N2*) as well as time frequency measures (Theta TSE, Theta ITPL, and Gamma TSE). Least Significant Difference (LSD) correction was used for multiple comparisons.

## 3 Results

### 3.1 Source localization

**Fig. 3:**
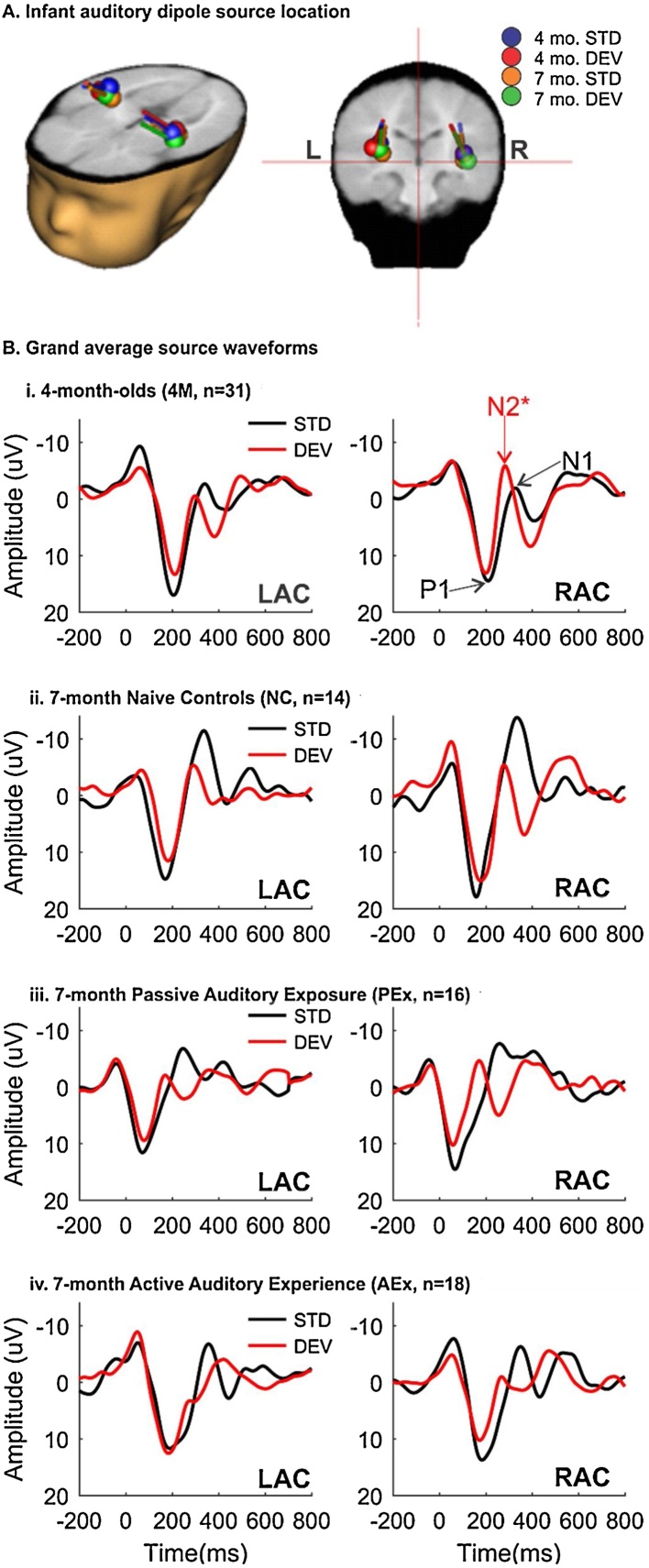
Left and Right Auditory Cortex (LAC, RAC) source locations and waveforms. A. LAC and RAC source localized dipoles are overlaid on the infant MRI template for 4- and 7-month old infants. For the 4-month group, standard (STD) stimulus is shown in blue and deviant (DEV) stimulus is shown in red. For the 7-month group, standard (STD) is shown in orange and deviant (DEV) is shown in green. Locations are not significantly different between 4 and 7 months-of-age. B. Grand average LAC and RAC source waveforms to the Standard, frequency invariant stimulus (STD, black) and the Deviant stimulus with rapid frequency change (DEV, red). STD Waveforms are characterized by a large positive peak ∼150 ms (P1), followed by a negative peak ∼350 ms (N1). P1 peak latencies and amplitudes to DEV stimuli are similar to STD across 4- and 7-month groups (i–iv). DEV stimuli elicit change complex (N2*), which changes in latency and morphology according to age and experience. (For interpretation of the references to color in this figure legend, the reader is referred to the web version of this article.)

Residual Variance of the two-dipole model fitting was less than 10% for the STD P1 peak (M_4M_ = 9.5%; M_NC_ = 9.2%; M_PEx_ = 9.7%, M_AEx_ = 9.1%), and slightly higher in the DEV condition (M_4M_ = 11.5%; M_NC_ = 9.8%; M_PEx_ = 13.5%, M_AEx_ = 10.3%). Fig. 3A shows dipole locations overlaid on the infant MRI template. At 4 and 7 months, STD and DEV grand average ERPs localize two dipoles in LAC and RAC. Following the fitting procedure, P1 and N1/N2* peaks were identified for each individual in the LAC and RAC source waveform, and measures of peak latency and amplitude were submitted to statistical testing. Fig. 3B shows grand

average source waveform morphology, and LAC/RAC peaks for 4- and 7-month-old Naïve Controls (3Bi, 3Bii), PEx (3Biii) and AEx infants (3Biv).

### 3.2 Cross sectional analysis between 4 and 7 month olds naïve infants: maturational effects

#### 3.2.1 Left and right auditory cortex source waveforms at 4 and 7 months

To assess age-related changes in the peak latency and amplitude of LAC and RAC source waveforms, mixed-design ANOVAs with age (4 month, 7 month) as a between-subjects factor and source (LAC, RAC) and condition (STD, DEV) as within-subjects factors were conducted with P1 and N1/N2* measures. The test of P1 latency revealed a main effect of age, *F*(1,34) = 20.91, *p* < 0.001. Table 1 shows that 7-month-olds had significantly faster P1 latencies for STD and DEV responses in both left and right auditory regions as compared to 4-month-olds, suggesting P1 peak latency decrease with age. No significant effects were found for P1 amplitude.

The test of N1/N2* measures revealed main effects of condition for latency, *F*(1,34) = 28.71, *p* < 0.001, and main effects of source for amplitude, *F*(1,34) = 10.83, *p* < 0.010. As expected, the N2*, which appears in the DEV condition only, had a shorter latency when compared to the N1 from the STD condition, at both ages (Table 1). In addition, the negative component was larger in the RAC as compared to LAC for both conditions and at both ages.

#### 3.2.2 Change in frequency magnitude and phase-coherence from 4 to 7 months

**Fig. 4:**
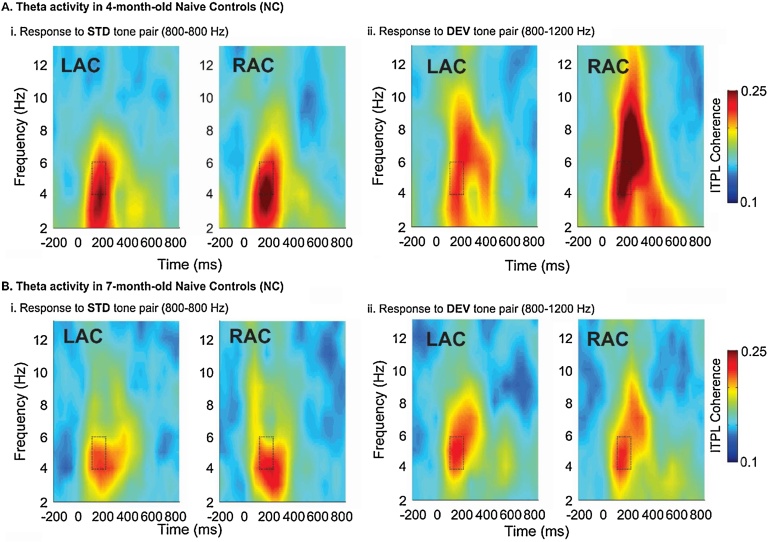
Inter-trial Phase Locking (ITPL) in 4- and 7-month-old infants to invariant standard (STD) and variant deviant (DEV) tone pairs in the Left (LAC) and Right (RAC) auditory cortices. Dashed-line boxes show significant time-frequency clusters. A) At 4-months-of- age, ITPL shows a low-frequency burst subsequent to first tone onset (time 0), to Standard (STD, Ai) and Deviant (DEV, Aii) tone pairs. Frequency change in the DEV condition (Aii) elicits a greater phase coherence in the RAC, compared to the LAC and the STD response. B) At 7 months this burst of phase-locked energy decreases and the rightward asymmetry in the DEV response (Bii) is lessened.

Maturation of frequency magnitude and phase-coherence over Left and Right auditory cortex was tested with the 2 × 2 × 2 mixed-design ANOVAs for *Theta ITPL, Theta TSE* and *Gamma TSE*. The test of *Theta ITPL* revealed main effects of age, *F*(1,34) = 7.150, *p* = 0.011, source, *F*(1,34) = 5.027, *p* = 0.032

and condition, *F*(1,34) = 26.461, *p* < 0.001, suggesting that the phase synchrony maturation from 4 to 7 months is different for STD and DEV stimuli. Fig. 4 shows the grand average *Theta ITPL* for 4- and 7-month-olds and illustrates that *Theta ITPL* decreases with age and is smaller at 7 months only in the DEV condition, and particularly in the RAC. Frequency change in the DEV condition elicits greater phase coherence in the RAC, compared to the LAC, particularly at 4 months of age. Thus it appears that the difference in maturation between conditions is largely due to the fact that RAC activity to DEV stimuli is very large at 4 months and then decreases in amplitude and shows bilateral activation at 7 months.

### 3.3 Auditory effect beyond maturation: group effects

#### 3.3.1 Left and right auditory cortex source waveforms in 7-month-old groups

P1 and N1/N2* modulation at 7-months-of-age due to auditory exposure was investigated by conducting 3 (group: AEx, PEx, NC) x 2 (source: LAC, RAC) × 2 (condition: STD, DEV) ANOVAs on P1 and N1/N2* latency and amplitude measures. Tests of N1/N2* revealed main effects of condition for latency *F*(1,37) = 38.897, *p* < 0.001 and amplitude −(1,37) = 8.531, *p* = 0.006. The N2* latency from the DEV condition was earlier and smaller as compared to the N1 from the STD condition for all three groups (Table 2).

#### 3.3.2 Changes in frequency magnitude and phase-coherence in 7-month-old groups

The mixed-design ANOVA for frequency magnitude and phase-coherence over Left and Right auditory cortex was used to assess source and condition differences across the 3 groups. A significant three-way interaction of group by source (LAC, RAC) by condition (STD, DEV) was observed for *Theta TSE*, *F*(2,44) = 9.825, *p* < 0.001. Two significant interaction effects in the simple ANOVAs were observed. First, the 2 (LAC, RAC) by 3 (AEx, PEx, NC) ANOVA was significant for Theta TSE in the STD condition, *F*(2,44) = 5.187, *p* = 0.009, suggesting a difference in lateralization across groups. Mean values, presented in Table 3, suggest that the interaction is driven by RAC differences between the AEx and NC groups. AEX infants had greater TSE magnitude in the RAC during STD processing, as compared to NCs. Mean TSE values in the PEx fell between the AEx and NC groups. Second, the 2 (STD, DEV) by 3 (AEx, PEx, NC) ANOVA was significant for Theta TSE in the RAC (*F*(2,44) = 6.422, *p* = 0.004), suggesting that the response to STD and DEV stimuli differed across groups. The means in Table 3 show that the AEx infants exhibited larger responses to the STD stimulus in the Right; whereas the NC and PEX infants

**Table 3:** Temporal Spectral Evolution in 7-month-old Infants with Varied Auditory Experience.

Cond.-Source	Theta Magnitude (% amplitude change)			Gamma Magnitude (% amplitude change)		
	NC	PEx	AEx	NC	PEx	AEx
	*M(SD)*	*M(SD)*	*M(SD)*	*M (SD)*	*M(SD)*	*M(SD)*
STD-R	9.7(5.6)	10.8(5.1)	13.1(6.7)	4.9(5.0)	6.0(7.1)	9.9(5.9)
STD-L	15.0(8.2)	12.0(4.4)	10.2(7.1)	6.8(5.9)	7.0(6.8)	3.4(6.9)
DEV-R	14.8(4.9)	12.8(7.7)	9.2(5.1)	4.2(6.8)	6.0(5.7)	6.5(6.8)
DEV-L	10.7(7.9)	11.0(7.0)	11.3(5.3)	2.1(6.3)	4.3(6.7)	9.2(6.6)

Cond: condition; STD: Standard; DEV: Deviant; R: right; L: left; %: percentage; NC: Naïve Controls; PEx: Passive.

**Fig. 5:**
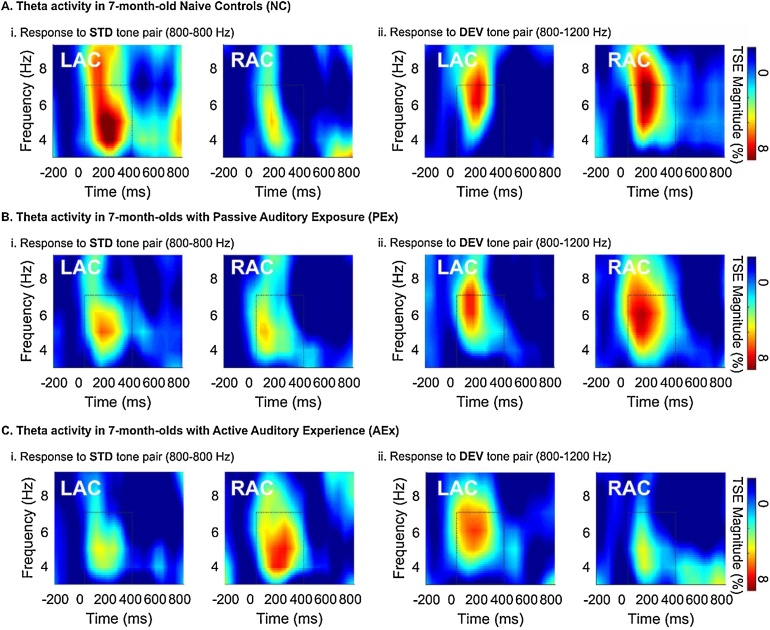
Grand Average Temporal Spectral Evolution (TSE) in 7-month-old infants to frequency-invariant standard (STD) and variant, deviant (DEV) tone pairs in the Left (LAC) and Right (RAC) auditory cortices. Dashed-line boxes show significant time-frequency clusters. A) Naïve Controls show clusters of TSE magnitude increase in the Theta band (3–7 Hz) to STD (Ai) and DEV (Aii) tone pairs. STD (frequency invariant) stimuli elicit a larger response in the LAC, compared to RAC. B) Infants with Passive Exposure show bilateral responses to both STD (Bi) and DEV (Bii) stimuli. C) Infants with Active Auditory Experience have greater magnitude in the RAC, compared to NCs. AEx infants also show a trend of greater response in the LAC, compared to RAC during DEV processing.

Exposure; AEx: Active Experience; M: mean; SD: Standard Deviation.

had larger responses to the STD stimuli in the LAC. Thus, it appears that sounds that do not change in frequency are processed in the right hemisphere in AEx infants, whereas sounds that change in frequency are processed in the left. Fig. 5 clearly illustrates this point, showing the grand average TSE magnitudes for 7-month old groups in the STD and DEV conditions. Fig. 5C shows the condition-specific pattern of processing seen in AEx infants, such that STD stimuli elicit a RAC > LAC pattern (Fig. 5Ci) and DEV stimuli (Fig. 5Cii) elicit a LAC > RAC pattern of Theta-band activity. NC (Fig. 5A) and PEx (Fig. 5B) do not show this strong condition-specific lateralization.

Analysis of *Gamma TSE* magnitude yielded a significant group by source by condition interaction effect, *F*(2,44) = 5.121 *p* = 0.010. A significant between-subjects effect was observed for Gamma TSE in the DEV condition in the 2 (LAC, RAC) by 3 (AEx, PEx, NC) ANOVA, *F*(2,44) = 4.721, *p* = 0.014), suggesting a group difference in the left hemisphere during DEV processing. In post-hoc comparisons of Gamma TSE in the LAC, only AEx and NC means differed significantly in the DEV condition (Mean Difference = 2.840, *p* = 0.016) with PEX means falling between the other two groups (Table 3).

**Fig. 6:**
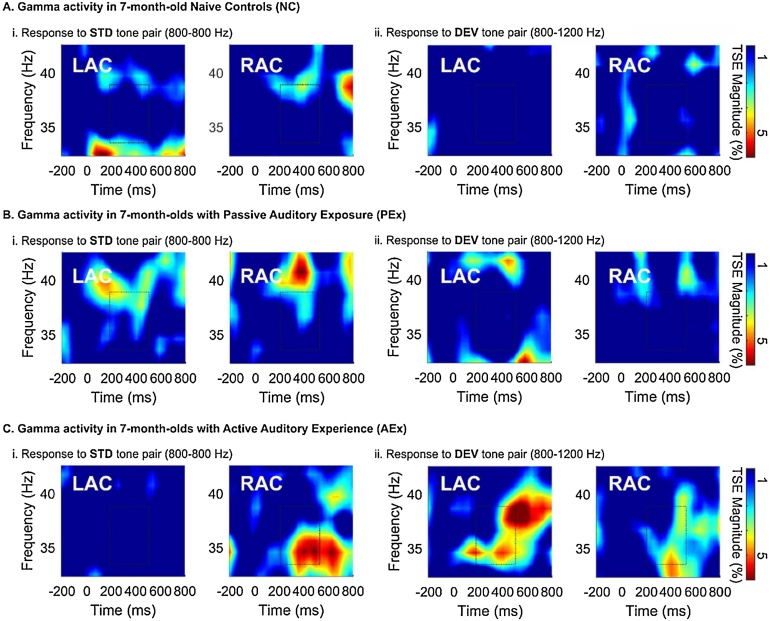
Grand Average Temporal Spectral Evolution (TSE) in 7-month-old infants to frequency-invariant standard (STD) and variant, deviant (DEV) tone pairs in the Left (LAC) and Right (RAC) auditory cortices. Dashed-line boxes show significant time-frequency clusters. A) Naïve Controls show small, scattered clusters of TSE magnitude increase in the Gamma band (33–37 Hz) to STD (Ai) and DEV (Aii) tone pairs. STD (frequency invariant) stimuli elicit somewhat larger response than DEV. B) Infants with Passive Exposure show a more coherent response than NCs, particularly to the STD stimuli (Bi). C) Infants with Active Auditory Experience have a clear Gamma cluster in the RAC to STD stimuli. LAC response to DEV stimuli is greater in AEx, compared to NC.

Fig. 6C illustrates that the condition-specific lateralization pattern observed in AEx infants over the Theta band is also observed in Gamma-band activity. Specifically, STD stimuli elicited a RAC > LAC pattern (Fig. 6Ci) and DEV stimuli (Fig. 6Cii) elicit a LAC > RAC pattern of Gamma-band TSE magnitude. NC and PEx show less activity overall in the Gamma band and again, do not appear to exhibit this more mature hemispheric weighting (Fig. 6A and B).

**Fig. 7:**
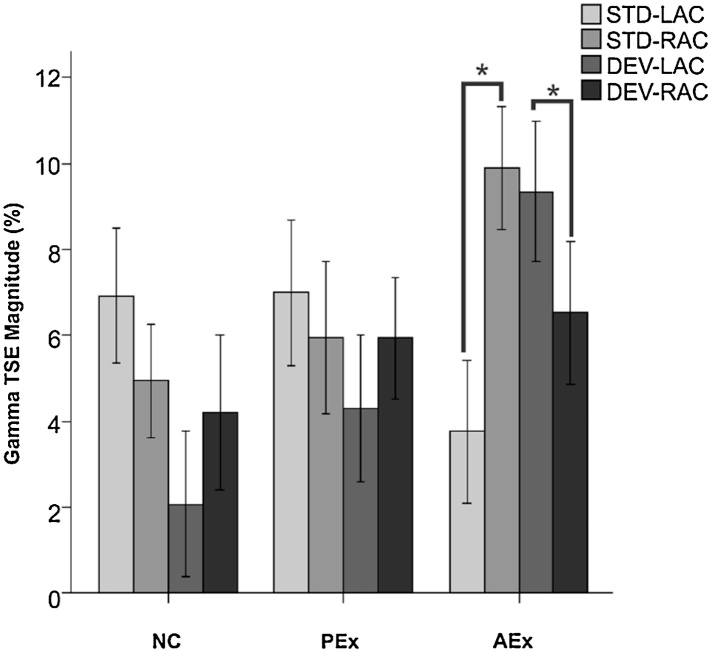
Mean Gamma-band (33–37 Hz) TSE magnitude to Deviant (DEV) tone pairs in Left and Right auditory cortices of 7-month-old infants with different levels of auditory experience. Infants with active auditory experience (AEx) have larger Gamma-band magnitude in the Left Auditory Cortex (LAC), compared to Naïve Controls (NC). Mean Gamma-band magnitude in infants with passive auditory exposure (PEx) is between NC and AEx, but not significantly different from either group. No group differences were observed in the Right Auditory Cortex (RAC).

Following examination of main effects, post-hoc paired *t*-tests were run with these variables to determine whether laterality of processing was specific to the AEx group. These tests showed that only the AEx infants showed significant differences between LAC and RAC for the STD (*t* = 2.329, *p* = 0.033) and DEV (*t* = 2.443, *p* = 0.027) conditions. No differences were observed between LAC and RAC in NC or PEx infants. Fig. 7 shows the mean values for TSE magnitude in the Gamma band for all groups highlighting the significant differences in AEx infants.

## 4 Discussion

The major findings of this study include: (1) increased speed of event-related source sensory response (P1 latency) and decreased Theta phase stability (ITPL) as a function of maturation from 4- to 7-months in NC infants; (2) the N2* peak on the deviant wave was shown to index specific modulation of frequency discrimination responses for both 4- and 7-month-old infants; and (3) changes in Theta and Gamma oscillatory dynamics emerged in the AEx group when compared to both PEx and NC groups.

### 4.1 Sensory response maturation from 4- to 7-months

P1 peak latencies from LAC and RAC sources decreased over maturation from 4- to 7-months in the naïve group in line with previous data showing faster sensory responses with increasing age (Ceponiene et al., 2002, Choudhury and Benasich, 2011). Source data also corresponded with the scalp-recorded ERP data from Benasich et al. (2014), showing faster P1 latencies in 7-month-olds, compared to 4-month-olds. An explanation of these findings could be increases in myelination of auditory system neurons and pathways or decreases in their synaptic density (Kinney et al., 1988, Su et al., 2008, Deoni et al., 2011).

If latency changes were associated with a decline in synaptic density, there should also be a decrease in peak amplitude, reflecting the response of a smaller number of active neurons. Although no significant peak amplitude differences were seen across maturation, a decrease in *Theta ITPL* was observed over the same time window as the P1 peak (i.e. 150–250 ms). Because ITPL is a measure of phase stability, the observed decrease in *Theta ITPL* suggests that as infants mature, processing efficiency and automatization may increase and thus less phase synchronization might be required to process auditory signals.

### 4.2 Frequency discrimination with maturation and acoustic exposure

The N2* component of the DEV response is of prime interest as it marks the beginning of the discrimination response and has been shown to be a robust infant predictor of later language outcomes (Benasich et al., 2006, Choudhury and Benasich, 2011). In Benasich et al. (2014), significant group differences manifested at several frontal, scalp-recorded electrodes showing faster N2* ERP latencies in the AEx and PEx groups as compared to naïve controls (NC). In the current study, effects of maturation and experience group on the DEV N2* latency and amplitude were non-significant. However, main effects of condition were observed at the N2* across all ages and groups. Taken together, the two studies suggest that the N2* peak on the deviant wave is indeed a robust marker of auditory discrimination and that auditory exposure can improve efficiency of acoustic processing. This hypothesis accords with animal models of plasticity, showing continuous modification of developing cortical representations by environmental cues (Kilgard et al., 2001, Woods et al., 2009, Froemke and Jones, 2011). Benasich et al. (2014) also reported increased N2* ERP amplitudes, higher P2 amplitudes and decreased P2 latencies to DEV stimuli in the AEx group, suggesting that attention to the training tones bestows a continuing processing advantage over and above the PEx group.

### 4.3 Enhanced response and lateralization of STD and DEV stimuli in AEx infants

Overall the AEx infants displayed more robust and precise acoustic mapping in Theta- and Gamma-band frequencies for both automatic sound processing and detection of rapid frequency change. Because this response is less clear in PEx and NC infants, we suggest that active engagement is an important catalyst for auditory plasticity at this age. This notion is akin to a multidimensional view of lateralization, which may vary over developmental epochs (Bishop, 2013) and matures in conjunction with learning in a rich and stimulating auditory environment (Minagawa-Kawai et al., 2011). In animal models, action potentials generated by cortical cells occur within the Gamma-band range of oscillations (Gray et al., 1989) and these responses can be enhanced with attention via phase-amplitude coupled modulation.

Our results also show that AEx infants show greater RAC response to STDs in the Theta band and greater LAC response to DEV stimuli in the Gamma band, as compared to NC infants. PEx infants do not differ from either AEx or NCs, and their mean values fall between the two groups for both conditions. These findings are compatible with the Asymmetric Sampling in Time theory (AST, Poeppel, 2003) which posits that the representation of acoustic stimuli in LAC and RAC is asymmetric, according to temporal divisions of the input into short (∼20–40 ms) and long (∼150–250 ms) temporal integration windows. Specifically, the AST suggests that the adult RAC preferentially encodes acoustic information over a long temporal integration window (e.g. envelope cues) by phase-reset and phase-locking in the Theta band, whereas the LAC extracts information that fluctuates over short time windows (e.g. fine-structure) via modulation of Gamma band activity. Our AEx group exhibits precisely this pattern, with robust Theta activity in the RAC during STD stimulation and robust Gamma activity in the LAC during DEV stimulation. The Naïve group showed the opposite pattern of activity in the Theta band (i.e. left lateralization during STD stimulation) and low or bilateral Gamma band activity in the DEV condition. This snapshot of longitudinal and cross-sectional infant data provides the basis for an exciting developmental arm of the AST hypothesis. Based on our data, we propose that the capacity of the auditory system to asymmetrically sample slow and fast acoustic information in the RAC and LAC, respectively, is both experience- and age-dependent in typically developing individuals. Furthermore, the data suggests while passive exposure increases automatic processing of auditory input, it is not sufficient to promote asymmetrical lateralization of oscillatory sampling in time. Lateralized, mature-like patterns, only emerge during attention-related interface with the acoustic information.

One hypothesis as to how this might be accomplished is related to the representation of spectral and temporal acoustic characteristics. Several lines of research have demonstrated lateralization of auditory processing based on spectral and temporal stability. In adults, rapid temporal dynamics, such as seen in speech sounds, are processed primarily in LAC and dense spectral dynamics in the RAC (Zatorre and Belin, 2001, Warrier et al., 2009). In our data, Theta-dominant processing in the RAC occurs in the STD condition when temporal dynamics dominate (i.e. two tones of the same F0 are presented in rapid succession) and Gamma-dominant processing occurs in the LAC during DEV stimulation when spectral changes are present (see Fig. 5). It may be that infants who have no previous or only passive exposure to these acoustic stimuli “focus” on stimuli temporal dynamics, whereas the AEx infants “focus” is on spectral features of the STD stimulus. This may signify a more “adult-like” treatment of sounds with a constant F0. An alternative hypothesis is that attention-based auditory experience cultivates an RAC-dominant asymmetry of Theta activity. Several studies suggest an intrinsic asymmetry across auditory cortices, such that Theta is dominant in RAC while Gamma dominates in LAC (Morillon et al., 2012, Hyafil et al., 2015). The two bands are thought to encode speech and sounds with speech-like timing via coordinated activity across hemispheres. Although the data presented here cannot distinguish between these two hypotheses, both suggest a more mature Theta encoding pattern for automatized processing of rapidly-presented speech-like sounds in AEx infants, compared to their NC and PEx counterparts.

The Gamma finding supports our conclusion of a “more mature pattern” of processing in AEx infants on several fronts. First, only the AEx group shows coherent activity in the Gamma range (see Fig. 6). In NC and PEx infants, Gamma-band TSE is low in power and irregular over time. Whereas all groups at this age show responses in the low-frequency Theta range, only the infants that had active auditory experience show a response in the high-frequency Gamma range. Studies have shown that the lower frequency bands emerge first during the development of oscillatory activity, and then activity gradually shifts toward higher frequency bands (Clarke et al., 2001, Koroleva et al., 2002, Marshall et al., 2002, Orekhova et al., 2006, Cragg et al., 2011, Ortiz-Mantilla et al., 2016). Thus, emergence of Gamma-band activity in the AEx infants, compared to NCs and PEx, suggests an advancement of high-frequency processing mechanisms beyond typical maturation. Emergence of Gamma oscillations in infants has also been linked to perceptual specialization for native phonemic contrasts at 6- (Ortiz-Mantilla et al., 2013), and particularly in the left hemisphere, at 12-months-of-age (Ortiz-Mantilla et al., 2013). The current data also suggest that sensitivity to rapid-frequency change, encoded in the Gamma band, may support emerging native phonemic specialization. Finally, it is important to consider that the greatest Gamma differences occur in LAC to DEV stimuli, with TSE magnitude larger in AEx infants compared to NCs. The DEV stimulus rapidly changes in F0 from 800 to 1200 Hz. Although this is a large frequency change, the timing of the change is rapid (70 ms ISI), approximating speech-like acoustic change. Therefore, LAC processing of DEV in the AEx group appears to accord with an adult-like LAC specialization for processing of rapid temporal dynamics. Together with the theta finding, our Gamma data support the notion of an emerging right/theta-left/gamma asymmetry in early development and in particular for responses to faster-rate stimuli.

### 4.4 Attention-driven neuroplasticity

The idea of attention-driven short- and long-term brain plasticity is not new. It has been proposed that even short-term auditory training with attentional modulation can retune neurons to segregate relevant sounds (Ahveninen et al., 2011). Repeated practice optimizes neuronal circuits by changing the number of neurons involved, the timing of synchronization and the number and strength of excitatory and inhibitory synaptic connections. Animals trained with specific auditory stimuli exhibit increased tonotopic organization of primary auditory cortex (Recanzone et al., 1993). Exposure to an immersive enriched acoustic environment, without training, enhances auditory cortical responses and sharpens tuning of auditory neurons, generalizing to unexposed sounds in both young and old animals (Engineer et al., 2004); enhancement can last for days with different time courses of decay for different peak components. It is therefore reasonable to suggest that infants who experience attention-driven auditory plasticity may exhibit changes in the number of neurons recruited for processing, increased strength of synaptic activity or enhanced definition of tonotopic map boundaries. This is especially important when we consider that at 4–7 months-of-age phonemic maps are being actively constructed (Ortiz-Mantilla et al., 2013). Therefore, active engagement with linguistic-like acoustic cues over this period has a good chance of impacting experience-dependent neuroplasticity and thus could bootstrap neuronal foundations critical to optimal language development.

## 5 Conclusions

Our results show that interactive auditory experience is associated with changes in oscillatory encoding and acoustic cortical mapping. We suggest that the ability of a child’s brain to automatically process and differentiate rapidly-changing acoustic cues may index neuronal maturation. Acoustic experience during the time when infants are constructing cortical maps for language appears to promote more mature oscillatory and lateralized patterns of rapid auditory processing and frequency discrimination. Further investigation is needed to determine longer-term outcomes of early interactive experience, and to directly test experience-dependent relationships among tone-pairs, speech encoding and language outcomes.

## Conflict of Interest

None.
